# Management of Acute Radiodermatitis in Non-Melanoma Skin Cancer Patients Using Electrospun Nanofibrous Patches Loaded with *Pinus halepensis* Bark Extract

**DOI:** 10.3390/cancers13112596

**Published:** 2021-05-26

**Authors:** Aikaterini Kyritsi, Stefanos Kikionis, Anna Tagka, Nikolaos Koliarakis, Antonia Evangelatou, Panagiotis Papagiannis, Alexandros Stratigos, Vangelis Karalis, Paraskevas Dallas, Andreas Vitsos, Efstathia Ioannou, Vassilios Roussis, Michail Rallis

**Affiliations:** 1 Section of Pharmaceutical Technology, Department of Pharmacy, School of Health Sciences, National and Kapodistrian University of Athens, Panepistimiopolis Zografou, 15784 Athens, Greece; katerinakyr18394@gmail.com (A.K.); vkaralis@pharm.uoa.gr (V.K.); dallas@pharm.uoa.gr (P.D.); avitsos@yahoo.gr (A.V.); 2 Section of Pharmacognosy and Chemistry of Natural Products, Department of Pharmacy, School of Health Sciences, National and Kapodistrian University of Athens, Panepistimiopolis Zografou, 15771 Athens, Greece; skikionis@pharm.uoa.gr (S.K.); eioannou@pharm.uoa.gr (E.I.); 3 First Department of Dermatology-Venereology, National and Kapodistrian University of Athens, Andreas Syggros Hospital, 5 Ionos Dragoumi Str., 11621 Athens, Greece; annatagka@gmail.com (A.T.); nkoliarakis@yahoo.gr (N.K.); antevagelatou@gmail.com (A.E.); PPaPagi@med.uoa.gr (P.P.); alstrat2@gmail.com (A.S.)

**Keywords:** acute radiodermatitis, radiation therapy, non-melanoma skin cancer, patients, *Pinus halepensis* bark extract, electrospun nanofibrous patches, anti-inflammatory activity

## Abstract

**Simple Summary:**

The most frequent adverse effect for patients receiving radiotherapy, an effective treatment for skin cancer when surgical removal of the tumor is impossible, is acute radiodermatitis, affecting patients’ physical function and often leading to therapy termination. Creams and other topical formulations used so far for the prevention of acute radiodermatitis are applied at regular intervals but do not ensure a constant and controlled transepidermal absorption. The aqueous extract of Aleppo pine bark, previously preclinically and clinically assessed in the form of gel, was herein loaded on micro/nanofibrous patches and clinically evaluated in comparison with a commercially used reference cream on non-melanoma skin carcinoma patients undergoing radiotherapy. The experimental patch significantly contributed to prophylaxis and successful management of acute radiodermatitis, safely restoring skin and its biophysical parameters to normal levels and reducing patients’ discomfort. Topical application of pine-loaded micro/nanofibrous patches holds great potential for the development of a new generation of anti-inflammatory skin care dressings against radiodermatitis.

**Abstract:**

Acute radiodermatitis is the most common side effect in non-melanoma skin cancer patients undergoing radiotherapy. Nonetheless, despite the ongoing progress of clinical trials, no effective regimen has been found yet. In this study, a non-woven patch, comprised of electrospun polymeric micro/nanofibers loaded with an aqueous extract of *Pinus halepensis* bark (PHBE), was fabricated and clinically tested for its efficacy to prevent radiodermatitis. The bioactivity of the PHBE patch was evaluated in comparison with a medical cream indicated for acute radiodermatitis. Twelve volunteer patients were selected and randomly assigned to two groups, applying either the PHBE patch or the reference cream daily. Evaluation of radiation-induced skin reactions was performed during the radiotherapy period and 1 month afterwards according to the Radiation Therapy Oncology Group (RTOG) grading scale, photo-documentation, patient-reported outcomes (Visual Analog Scale, questionnaire), biophysical measurements (hydration, transepidermal water loss, erythema, melanin), and image analysis. In contrast with the reference product, the PHBE patch showed significant anti-inflammatory activity and restored most skin parameters to normal levels 1 month after completion of radiation therapy. No adverse event was reported, indicating that the application of the PHBE patch can be considered as a safe medical device for prophylactic radiodermatitis treatment.

## 1. Introduction

Skin cancer is one of the most common malignancies, constituting an important public health concern [1]. Non-melanoma skin cancer (NMSC) includes mainly basal cell carcinoma (BCC), squamous cell carcinoma (SCC), and basosquamous carcinoma (BSC) with features of both basal cell and squamous cell carcinomas [2,3,4,5,6]. The factors that are implicated in NMSC etiopathogenesis include chronic ultraviolet (UV) radiation exposure, phototype, age, gender, immunosuppression, smoking, and genetic factors [1,7,8,9]. NMSC is relatively non-lethal and non-invasive, while its incidence varies among race and region. It mainly occurs on the head and neck, anatomical sites which are frequently treated with X-ray therapy [4,7,8].

Radiation therapy (RT) is an effective alternative treatment when surgical removal is contraindicated due to the tumor’s anatomical site or patient comorbidities [8,10]. Orthovoltage X-ray therapy is considered as one of the traditional approaches for treating skin cancer and covers an X-ray energy range of 150–300 kV, damaging DNA cancer cells either directly or through free radical generation [11,12]. However, as with other cancer treatments, RT is also associated with side effects [13]. Acute radiodermatitis is the most frequent adverse effect in patients undergoing radiotherapy [14]. Its severity depends on the dose per fraction, the total dose, and the individual’s sensitivity [13,14,15,16]. Onset of acute radiodermatitis may occur between 15 days to 3 months after the beginning of the treatment and ranges from faint erythema to dry desquamation, moist desquamation, and ulceration [13,15,17]. Radiation-induced skin injury may impair the patient’s physical functioning and compliance with therapy [17,18].

Clinical practice guidelines for the prophylaxis and management of acute radiodermatitis include a variety of topical, oral, and intravenous agents [14,19]. Surveys suggest that topical products, such as creams, gels, and lotions, should be used to protect and promote tissue repair in patients with radiation-induced dermatitis [19,20]. The main disadvantage of the application of such products is the non-controlled dosage, which leads to incomplete preventive and therapeutic effects [19]. Numerous topical formulations have been clinically studied in radiation-induced skin injuries without providing sustainable treatment strategies [14,18,19,21].

The advantage of topical patches, which have yet to be evaluated for the management of radiodermatitis in NMSC patients, may be the controlled delivery of anti-inflammatory agents [22,23,24,25,26]. The inflamed area and surrounding skin can be effectively protected against microbial contamination by biocompatible composite dressings [27,28,29].

Over the last few years, nanotechnology has risen as one of the most promising technologies in the development of patches for biomedical applications. Nanofibrous non-wovens are steadily attracting increasing interest for their application as wound dressings, controlled drug release systems, and tissue regeneration scaffolds [29,30,31,32]. Exhibiting high surface-area-to-volume ratio, high porosity, and tunable mechanical strength, electrospun fibers with a size in the micro/nanoscale can be easily generated through electrically charged polymeric solutions or melts [33,34]. Under the application of a high-voltage electric field, various synthetic and natural polymers or blends can be fabricated in micro/nanofibrous matrices with a structural similarity to the natural extracellular matrix, affording high drug loading efficacy and displaying tunable mechanical properties. Moreover, the incorporation of suitable bioactive substances into the polymeric fibers of various biodegradable and biocompatible polymers can lead to multifunctional topical dressings with anti-inflammatory properties [35,36,37,38,39].

Species of the genus *Pinus* are well known for their medicinal properties, which are related to their chemical composition. Their cones, needles, and bark extracts, as well as their essential oils, have been utilized for many pharmaceutical applications, demonstrating cytotoxic, analgesic, antiviral, antioxidant, antimicrobial, and/or anti-inflammatory activities [40,41,42,43,44,45]. In the Mediterranean region, *Pinus halepensis* Miller (Aleppo pine) represents one of the most common naturally growing conifer species. Its aqueous bark extract (PHBE) is rich in antioxidant polyphenolic agents, consisting mainly of procyanidins and phenolic acids.

Herein, motivated by the promising results of our previous studies, which showed that PHBE can significantly prevent and/or decrease skin damage caused by UV radiation or X-ray irradiation [16,26,46], non-woven polymeric micro/nanofibrous patches loaded with PHBE were prepared and clinically evaluated for their ability to prevent acute radiodermatitis in NMSC patients undergoing radiotherapy (Figure S1). The PHBE micro/nanofibrous patch was morphologically and physicochemically characterized by scanning electron microscopy (SEM), as well as thermogravimetric (TGA) and differential scanning calorimetry (DSC) analyses. The efficiency of the micro/nanofibrous PHBE dressing was evaluated in comparison with a medical cream indicated for acute radiodermatitis. The clinical evaluation was conducted on 12 volunteer patients randomly assigned to two groups, applying either the PHBE patch or the reference cream daily. The radiation-induced skin reactions were evaluated during the period receiving radiotherapy and 1 month afterwards according to the Radiation Therapy Oncology Group (RTOG) grading scale, photo-documentation, patient-reported outcomes (Visual Analog Scale (VAS), questionnaire), measurements of the skin’s biophysical parameters (hydration, transepidermal water loss (TEWL), erythema, melanin), and image analysis.

## 2. Materials and Methods

### 2.1. Materials

Polyethylene oxide (PEO) (MW 8,000,000) and cellulose acetate (CA) (MW ~50,000) were purchased from Sigma-Aldrich (Darmstadt, Germany). Sodium alginate (SA) (MW 216,121) was purchased from Cellco Chemicals SA. *P. halepensis* bark was collected from Kaisariani forest, a suburb near Athens, Greece, and pulverized in a blender. The bark was extracted with dH_2_O (1:10 ratio for 48 h at 40 °C) and subsequently filtered and freeze-dried to produce a dark red powder. All chemical reagents used were of analytical grade.

### 2.2. Preparation of the Electrospun Micro/Nanofibrous Patch

The PHBE micro/nanofibrous patches were prepared by electrospinning CA/PHBE and PEO/SA spinning solutions as previously described, with slight modifications [26]. For preparation of the CA/PHBE spinning solution, CA at 9% *w*/*v* and PHBE at 2% *w*/*v* were dissolved in Me_2_CO/H_2_O (9:1 *v*/*v*). The PEO/SA solution was prepared in H_2_O by dissolving PEO at 1.5% *w*/*v* and SA at 3% w/v. Both spinning solutions were prepared at room temperature under stirring for 24 h to ensure their homogeneity. The polymer solutions were electrospun from 10 mL disposable syringes fitted with 23G tip-ground-to-flat needles. The syringes were mounted on two Harvard PHD 2000 programmable syringe pumps (Harvard Apparatus, Holliston, MA, USA), which were positioned horizontally on an antiparallel setup to ensure the homogeneous blending of the fibers. Electrospinning was performed using a γ-High Voltage Research DC power supply generator with a maximum voltage of 50 kV (Gamma High Voltage Research, Ormond Beach, FL, USA). The applied voltage was fixed at 25 kV. For the CA/PHBE solution, the tip-to-collector distance was fixed at 10 cm, whereas for the PEO/SA solution, the distance was fixed at 20 cm. The CA/PHBE solution feeding rate was fixed at 2 mL/h, while the PEO/SA solution feeding rate was adjusted at 0.5 mL/h, resulting in a 4:1 (*w*/*w*) blending ratio of CA/PHBE fibers to PEO/SA fibers, with a 16.5% *w*/*w* concentration of PHBE in the fabricated matrices. The produced nanofibers were collected on an RC-6000 (NaBond Technologies, Hong Kong) rotating drum collector wrapped with aluminum foil, at a rotation speed of 400 rpm. Temperature and relative humidity were 20 ± 2 °C and 60 ± 5%, respectively.

### 2.3. Characterization of the Micro/Nanofibrous Patch

A desktop PhenomWorld scanning electron microscope (Thermo Fischer Scientific, Waltham, MA, USA) with a charge reduction sample holder and tungsten filament (10 kV) was used for the morphological characterization of the micro/nanofibers of the PHBE patch. To determine the average diameter of the fibers, the diameters of 100 fibers from each SEM image were measured in the embedded image analysis software (Phenom Pro Suite/Fibermetric). TGA analysis was conducted using a TA Thermogravimetric Analyzer (TGA 55, TA Instruments, New Castle, DE, USA) at a 10 °C/min heating rate from 40 to 600 °C under a 25 mL/min nitrogen flow. Sample weight, sample temperature, and heat flow were recorded continuously. DSC analysis was performed using a TA Thermal Analyzer (Discovery DSC 25, TA instruments, New Castle, DE, USA). Samples of 6–7 mg sealed in aluminum pans were heated from 40 to 300 °C at a constant rate of 10 °C/min under a 25 mL/min nitrogen flow.

### 2.4. Study Design and Patient Selection

All performed procedures were carried out in accordance with the Good Clinical Practice (GCP) guidelines established by the Directive 2001/20/EC, the Federal Code of Users of USA (21 CFR Part 312), and the International Conference on Harmonization (ICH). The study was conducted in accordance with the principles of the Declaration of Helsinki (Directive 2001/83/EC; ICH Issue E9 1996; Directive 2001/20/EC; Directive 2002/98/EC; Directive 2003/63/EC; ICH E (6) R1; 21 CFR Part 312; WHO 2008).

The volunteers were selected among the Radiotherapy Oncology Department patients of Andreas Syggros Hospital from November 2019 to February 2020. Eligible patients were adults with histologically confirmed NMSC before receiving radiation therapy. NMSC was located in the scalp, nose, lip, forehead, cheek, alar nasal sulcus, and nail. The exclusion criteria were pregnancy, breastfeeding, concomitant chemotherapy, immunosuppressive treatment, previous radiotherapy to the treated area, and patients with other autoimmune skin diseases, including atopic dermatitis, psoriasis, and ichthyosis.

This was an open-label 2-treatment clinical research trial comparing the efficacy of a new PHBE patch versus the reference cream. Twelve patients were selected and randomly assigned to 2 groups, applying either a PHBE patch or a reference medical cream product daily. All patients were patch tested for hypersensitivity to the PHBE patch and the reference cream’s components.

### 2.5. Radiotherapy Schedules

The patients received local conventional radiotherapy (CRT) of 250 cGy in 23 fractions to a total dose of 5750 cGy 5 times a week with slight modifications to schema therapy depending on the case. The radiation energy was generated by orthovoltage X-rays (Xstrahl 200, Walsall, UK).

The patients were instructed to apply to the irradiated area either a PHBE patch for 24 h every day or a thin layer of the reference cream twice a day during the radiotherapy period and for 1 month after its completion.

### 2.6. Clinical Assessment

All enrolled patients completed the study. Their mean age was 80 years; 75% were men and 25% were women. Among the patients, 50% had BCC, 25% had SCC, and 25% had BSC. Among all patients, 83.3% were surgically treated before receiving RT; 58.3% had primary and 25% had recurrent NMSC. Half of the patients received the micro/nanofibrous patch loaded with *P. halepensis* bark extract and the other half of the patients received the commercially available medical cream (reference product).

For all patients, a full medical history and demographic data, including age, body mass index (BMI), phototype (Fitzpatrick skin type), medical diseases, family history, sun exposure, and smoking, were recorded (Table 1).

The most important diagnostic criteria for successful prophylaxis and treatment of acute radiodermatitis included clinical estimation and photo-documentation.

Radiation dermatitis on the irradiated skin was assessed according to the Radiation Therapy Oncology Group (RTOG) Common Terminology Criteria for Adverse Events (CTCAE) version 4.0 [16] during Days 7, 14, 21, and 28, and 1 month after RT (Day 60).

Skin inflammation was recorded before RT (Day 0), during Days 7, 14, 21, and 28, and 1 month after RT (Day 60). Skin images were acquired using a Nikon D5100 digital camera (Nikon, Tokyo, Japan) equipped with an AF-S Micro Nikkor 60 mm f/2.8 G ED lens (Nikon, Tokyo, Japan), which was at a distance of 33 cm perpendicular to the skin.

Radiation-associated symptoms of pain and itching were reported by patients during Days 7, 14, 21, and 28, and 1 month after RT (Day 60) using a visual analogue scale (VAS) (10 cm in length; 0 = no symptoms, 10 = highly intense symptoms).

### 2.7. Skin Analysis

The skin was evaluated by an Antera 3D camera (Miravex, Dublin, Ireland) before RT (Day 0), during Days 7, 14, 21, and 28, and 1 month after RT (Day 60). Hemoglobin concentration and skin texture were assessed with Antera 3D software (Miravex, Dublin, Ireland).

### 2.8. Measurements of the Skin’s Biophysical Parameters

Skin parameters, including hydration, TEWL, erythema, and melanin, were evaluated using non-invasive biophysical methods before RT (Day 0), during Days 7, 14, 21, and 28, and 1 month after RT (Day 60). Hydration was measured using a Corneometer CM 820 (Courage + Khazaka electronic GmbH, Köln, Germany) through changes in the dielectric constant. The indications were recorded in arbitrary units. The barrier function of the skin (TEWL) was evaluated using a Tewameter TM 210 (Courage + Khazaka electronic GmbH, Köln, Germany) by measuring the density gradient of the water evaporation from the skin. The estimation was based on the mean value of the flux density of water (in g/m^2^/h), which was obtained 1 min after the beginning of the measurement. Erythema and melanin were calculated using a Mexameter MX 18 (Courage + Khazaka electronic GmbH, Köln, Germany) by measuring absorption/reflection at 3 different light wavelengths. The indications were recorded in arbitrary units. Before each measurement, the treated area was cleaned with 0.9% sodium chloride solution and wiped with sterile gauze.

### 2.9. Patient Self-Report

The two therapeutic interventions were evaluated by patients using a questionnaire 1 month after RT (Day 60). The color, texture, applicability, ease of use, absence of irritation, and overall evaluation of the PHBE patch and the reference product were assessed using a standardized scale from 0 (maximum negative impact) to 5 (maximum positive impact).

### 2.10. Statistical Analysis

Statistical analyses were performed using the Statistical Package for the Social Sciences (SPSS version 25, IBM, Chicago, IL, USA). Data were described using the mean ± standard deviation (SD) and proportions. A normality test was conducted to determine whether the sample data had been drawn from a normally distributed population based on Kolmogorov–Smirnov and Shapiro–Wilk criteria. Either an independent sample *t*-test for parametric measurements or the Mann–Whitney U-test for non-parametric measurements was used to calculate differences between the therapeutic interventions. Treatment values were compared with the pretreatment baseline by the parametric method of a paired *t*-test or the non-parametric Wilcoxon-signed rank test method in both treatments; *p*-values of < 0.05 were considered statistically significant and are indicated by the (*) symbol for *p* < 0.05, (**) for *p* < 0.01, (***) for *p* < 0.001, and (****) for *p* < 0.0001.

## 3. Results and Discussion

Polymeric micro/nanofibers loaded with PHBE were obtained as a non-woven patch and evaluated for their ability to prevent acute radiodermatitis in patients with NMSC undergoing radiotherapy. Based on our previous findings [24], SA, PEO, and CA polymers were selected for the fabrication of the PHBE micro/nanofibrous patches, since they are considered to be non-toxic biopolymers of choice for many wound dressings and other biomedical applications [47,48,49].

**Figure 1:**
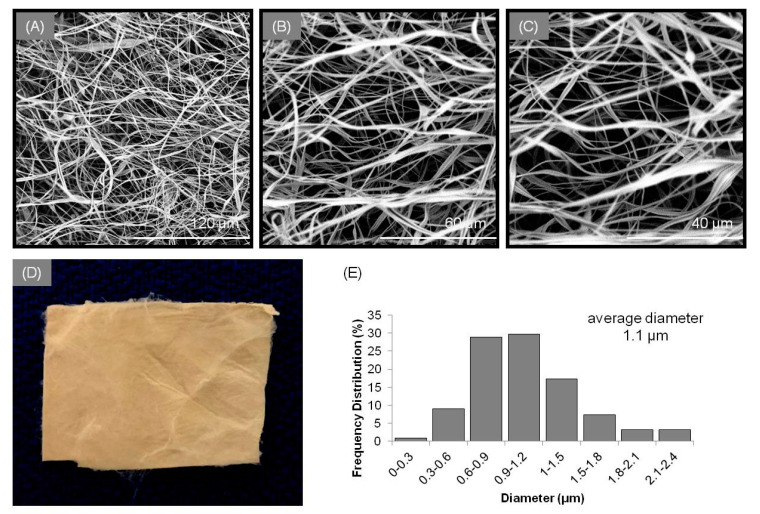
SEM images of the PHBE micro/nanofibrous patch at (**A**) 1000×, (**B**) 2000×, and (**C**) 3000× magnification. (**D**) Image of a PHBE micro/nanofibrous patch. (**E**) Average diameter distribution histogram of fibers in PHBE dressings.

The simultaneous electrospinning of the CA/PHBE and PEO/SA spinning solutions on the same rotating drum collector through an antiparallel electrospinning setup ensured the successful blending of the fibers in the patches. By fine-tuning the electrospinning parameters [26], a uniform fibrous mat was obtained with bead-free fibers. Analysis of the SEM images (Figure 1) revealed blended fibers of ribbon-like and cylindrical morphologies with size diameters ranging from 289 nm to 2.4 μm and an average diameter size of 1.1 ± 0.22 μm.

**Figure 2:**
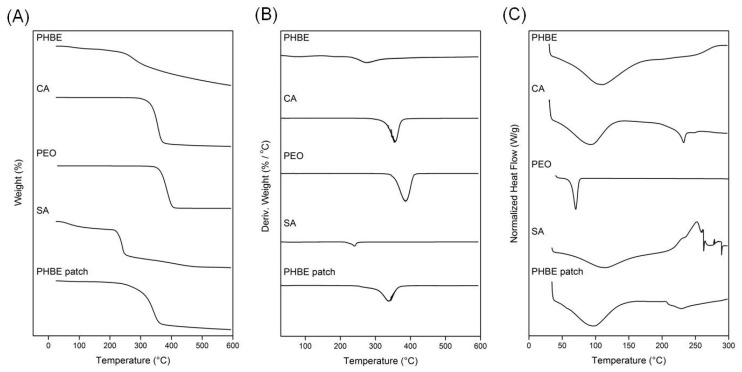
(**A**) TGA, (**B**) DTG and (**C**) DSC thermograms of the raw materials and the electrospun PHBE micro/nanofibrous patch.

The fabricated PHBE micro/nanofibers were physicochemically characterized by TGA and DSC analyses. In the TGA thermograms (Figure 2A), all starting materials (PHBE, CA, PEO, and SA powders) and the electrospun PHBE micro/nanofibrous patch recorded a single degradation step. PHBE started to decompose at 251 °C, the degradation step of CA was recorded at 322 °C, PEO decomposition initiated at 352 °C, and SA showed a decomposition step at 216 °C. The initial slight mass loss in the case of PHBE and SA is attributed to the volatilization of moisture and hydrogen-bound water. The PHBE fibers showed a broad thermogravimetric curve with a slight mass loss due to moisture volatilization up to their decomposition step at 272 °C. The different thermal behavior of the PHBE micro/nanofibrous patch was also evident in the derivative thermogravimetry (DTG) thermograms (Figure 2B). The maximum decomposition rate was recorded at 275 °C for PHBE, 355 °C for CA, 385 °C for PEO, 240 °C for SA, and at 338 °C for the PHBE fibers.

In the DSC thermograms (Figure 2C), the broad endotherms below 100 °C recorded for PHBE, CA, and SA are attributed to dehydration phenomena. CA and PEO showed melting endothermic peaks at 232 °C and 70 °C, respectively, whereas the exothermic bands of SA and PHBE at approximately 250 °C are associated with degradation events. The PHBE micro/nanofibrous patch showed a broad dehydration endotherm between the ambient temperature and 100 °C, followed by an endothermic melting band at 228 °C and a broad degradation exotherm over 250 °C, revealing a different thermal profile arising from the synergistic degradation events of the combined raw materials.

According to the characteristics of the patients (Table 1), there were no significant differences between the group of patients receiving the PHBE patch therapy (PHBE patch patients) and the group of patients receiving the reference cream therapy (reference cream patients) concerning the demographics, tumor characteristics, and RT doses (*p* > 0.05) of the patients (Table 2).

**Figure 3:**
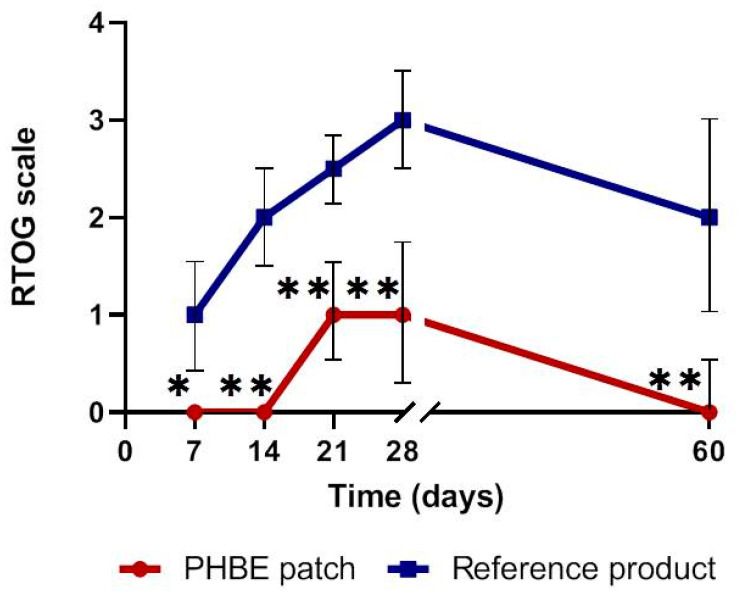
Clinical estimation of acute radiodermatitis based on RTOG criteria, before radiation therapy (RT) (Day 0); during Days 7, 14, 21, and 28; and 1 month after RT (Day 60). Significant differences were observed between the two interventions throughout the treatment period (* *p* < 0.05, ** *p* < 0.01). Total skin recovery was observed in the PHBE patch patients 1 month after RT (Day 60).

The clinical estimation of acute radiodermatitis based on RTOG criteria is presented in Figure 3. In PHBE patch patients, acute radiodermatitis onset was observed after 21 days and reached a maximum Grade 1 on the RTOG scale, while in the patients using the reference cream, the onset was observed after 7 days, reaching a maximum Grade 3. More specifically, in the first 2 weeks of RT, no radiodermatitis was observed in the PHBE patch patients, while all reference cream patients had Grade 1–2 acute radiodermatitis. In the next 2 weeks, the PHBE patch patients had Grade 1–2 radiodermatitis, while the reference cream patients had Grades 2–3. One month after completion of RT (Day 60), only PHBE patch patients displayed total skin recovery without signs of radiodermatitis, while 50% of the reference cream patients had Grade 1 acute radiodermatitis, 17% had Grade 2, and 33% had Grade 3. The acute radiodermatitis incidence was significantly different between the two interventions throughout the whole study period (Day 7, *p* = 0.015; Day 14, *p* = 0.002; Day 21, *p* = 0.002; Day 28, *p* = 0.009; Day 60, *p* = 0.004).

**Figure 4:**
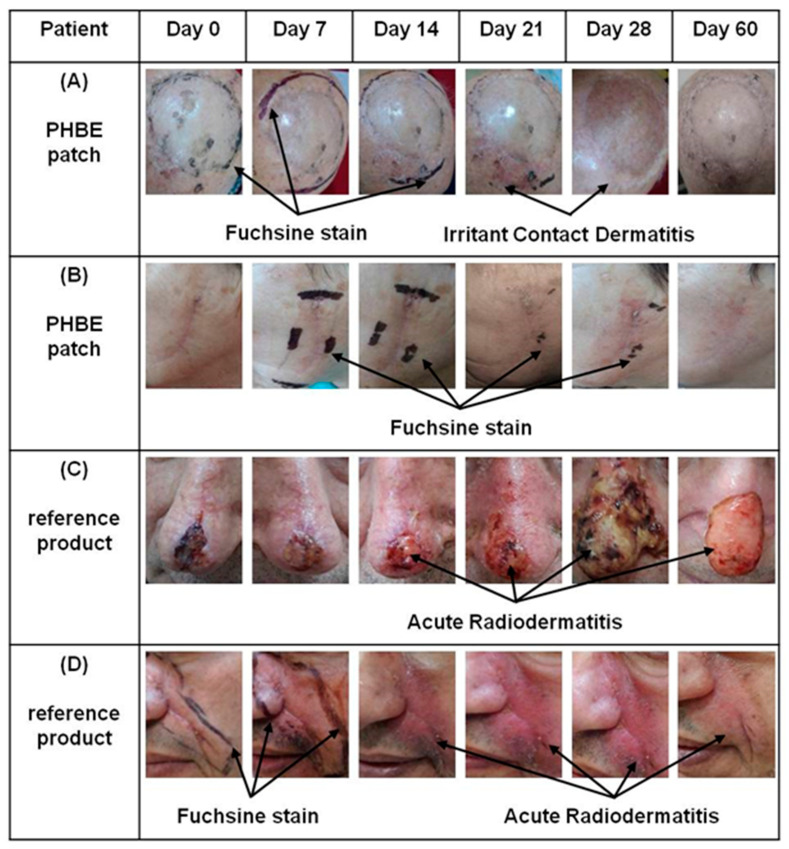
Representative images of (**A**) an 81-year-old male patient treating a scalp transplant with a PHBE patch, (**B**) an 80-year-old female patient treating a cheek with a PHBE patch, (**C**) an 82-year-old male patient treating the nose and nasal tip with the reference product, and (**D**) a 58-year-old male patient treating the cheek and alar nasal sulcus with the reference product before RT (Day 0); during Days 7, 14, 21, and 28; and 1 month after RT (Day 60). In contrast to the reference cream, the PHBE patch demonstrated significant anti-inflammatory efficacy.

The above results were confirmed by photo-documentation (Figure 4, Figures S2 and S3). Clinical evaluation showed that the PHBE patch significantly soothed inflamed skin, preventing and treating acute radiodermatitis. No clinical signs of acute radiodermatitis were observed 1 month after completion of RT (Day 60) in the PHBE patch patients (Figure 4A,B). Only one patient developed irritant contact dermatitis due to the medical tape, which was treated by application of a PHBE patch on the irritated area (Figure 4A, Days 21 and 28). In the case of the reference cream, extended inflammation was observed for all patients that suffered mechanical skin injuries due to the difficulty in cream removal (Figure 4C,D).

**Figure 5:**
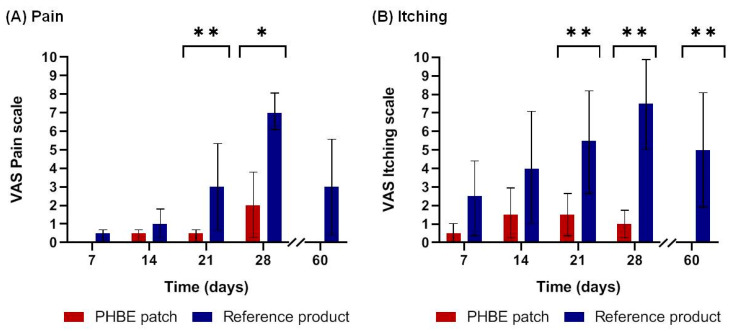
Patient self-report of (**A**) pain and (**B**) itching based on a visual analog scale (VAS) before radiation therapy (RT) (Day 0); during Days 7, 14, 21, and 28; and 1 month after RT (Day 60). Significant differences were observed between the two interventions throughout the treatment period (* *p* < 0.05, ** *p* < 0.01). In contrast to the reference cream patients, no pain or itching was observed in the PHBE patch patients 1 month after RT (Day 60).

Mean pain and itching were significantly lower for patients applying the PHBE patch in comparison with those applying the reference cream. No pain or itching were observed 1 month after RT (Day 60) only in PHBE patch patients (Figure 5). In total, 73% of patients using the PHBE patch completed the study pain-free, while all patients using the reference cream suffered from pain. The mean pain scores were higher in the reference cream patients. Significant differences were recorded between the two interventions (Figure 5A) (Day 21, *p* = 0.009; Day 28, *p* = 0.03). No itching was observed 1 month after completion of RT (Day 60) for patients using the PHBE patch, while all patients using the reference cream showed persistent itching. The mean itching scores were higher in the reference cream patients. Significant differences were recorded between the two interventions (Figure 5B) (Day 21, *p* = 0.004; Day 28, *p* = 0.004; Day 60, *p* = 0.002).

**Figure 6:**
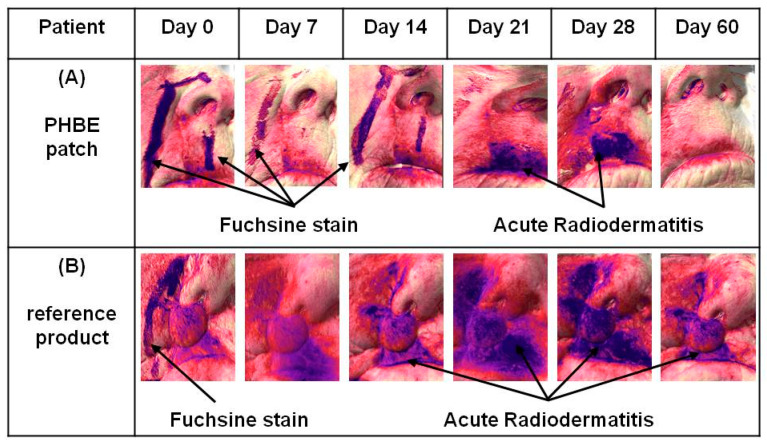
Representative Antera 3D images of (**A**) an 87-year-old female patient treating the alar nasal sulcus and upper lip area with a PHBE patch and (**B**) an 85-year-old female patient treating the alar nasal sulcus and upper lip area with the reference product before radiation (RT) (Day 0); during Days 7, 14, 21, and 28; and 1 month after RT (Day 60). In contrast to the reference cream, the PHBE patch demonstrated significant anti-inflammatory efficacy and skin recovery 1 month after RT (Day 60).

According to the images analyzed with Antera 3D software (Figure 6, Figures S4 and S5), the PHBE patch exhibited remarkable anti-inflammatory activity on radiation-exposed skin and contributed to the recovery process 1 month after completion of RT (Day 60). The reference cream showed rapid onset, progression, and failure in treating acute radiodermatitis.

**Figure 7:**
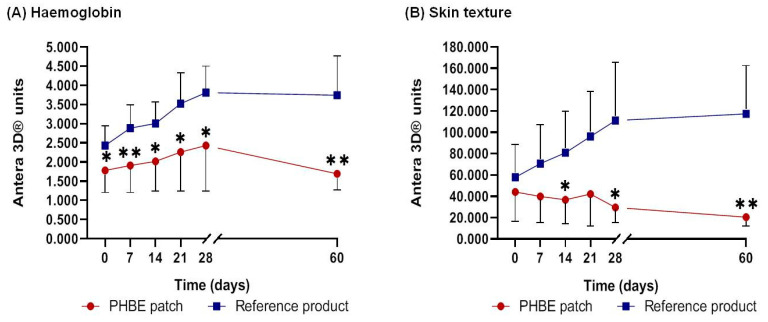
(**A**) Hemoglobin concentration and (**B**) skin texture value before radiation therapy (RT) (Day 0); during Days 7, 14, 21, and 28; and 1 month after RT (Day 60). Significant differences were recorded between the two interventions at most of the time points (* *p* < 0.05, ** *p* < 0.01). Hemoglobin concentration and skin texture value returned to the initial levels 1 month after RT (Day 60) in patients using the PHBE patch.

Mean hemoglobin concentration and skin texture value increase when inflammation intensity escalates. A lower rate of hemoglobin concentration was observed throughout the study period for patients using the PHBE patch in comparison with the patients using the reference cream. One month after completion of RT (Day 60), hemoglobin concentration returned to the initial levels for the PHBE patch patients, while it remained increased for patients using the reference cream. Significant differences were recorded between the two interventions throughout the treatment period (Figure 7A) (Day 0, *p* = 0.016; Day 7, *p* = 0.006; Day 14, *p* = 0.013; Day 21, *p* = 0.025; Day 28, *p* = 0.047; Day 60, *p* = 0.002). A significant difference was observed between Day 0 and Day 60 in the reference cream patients (*p* = 0.002).

A lower value of skin texture was measured throughout the treatment period for patients using the PHBE patch in comparison with the patients using the reference cream. After Day 21, skin texture returned to the initial values for PHBE patch patients, while it increased for patients using the reference cream. Significant differences were recorded between the two interventions throughout the treatment period (Figure 7B) (Day 14, *p* = 0.045; Day 28, *p* = 0.027; Day 60, *p* = 0.004). A significant difference was observed between Day 0 and Day 60 in both intervention patients (PHBE patch, *p* = 0.037; reference creams *p* = 0.005).

**Figure 8:**
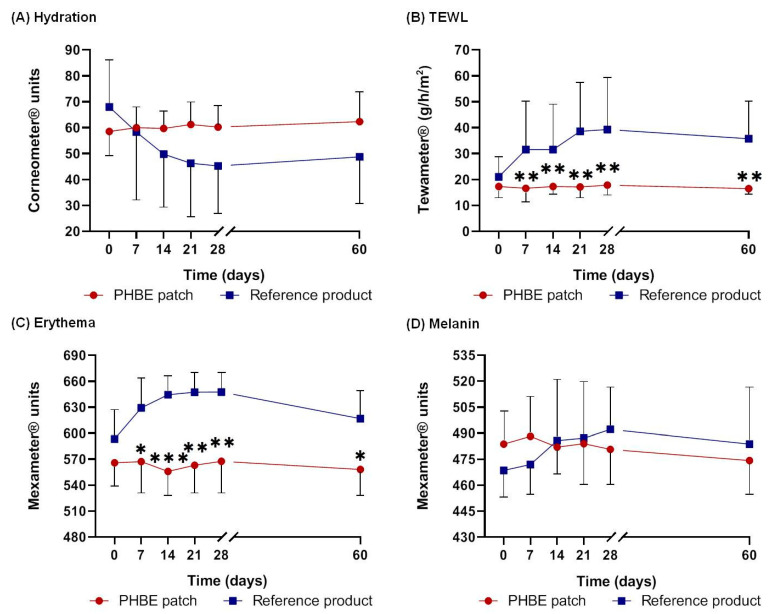
Evaluation of (**A**) hydration, (**B**) TEWL, (**C**) erythema, and (**D**) melanin, before radiation therapy (RT) (Day 0); during Days 7, 14, 21, and 28; and 1 month after RT (Day 60). Regarding TEWL and erythema, significant differences were recorded between the two interventions at most of the time points (* *p* < 0.05, ** *p* < 0.01, *** *p* < 0.001). All the biophysical skin parameters returned to the initial values 1 month after RT (Day 60) for the patients using the PHBE patch.

Hydration, TEWL, and erythema, as well as melanin to a lesser degree, are directly associated with inflammation severity [27]. Upon skin inflammation, TEWL, erythema, and melanin levels increase, whereas hydration decreases. All measurements of biophysical skin parameters showed that the PHBE patch was able to re-establish a normal skin barrier and return the stratum corneum to its physiological state 1 month after RT (Day 60) (Figure 8).

The PHBE patch maintained the hydration levels of the stratum corneum at normal values, while the application of the reference cream resulted in lower hydration of the skin. However, no significant difference was observed between the two interventions at any time point (Figure 8A).

Before RT (Day 0), the skin barrier function was similar in the two interventions. From Day 7 to Day 60, TEWL remained almost stable for the patients using the PHBE patch, while it continuously increased for those using the reference cream. Significant differences were observed between the two interventions at most of the time points (Figure 8B) (Day 7, *p* = 0.004; Day 14, *p* = 0.004; Day 21, *p* = 0.002; Day 28, *p* = 0.004; Day 60, *p* = 0.002). A significant difference between Day 0 and Day 60 was recorded for the patients using the reference product (*p* = 0.043).

A lower value of erythema was observed throughout the study period for the PHBE patch patients compared with the patients using the reference cream. Significant differences were recorded between the two interventions for most of the time points (Figure 8C) (Day 7, *p* = 0.017; Day 14, *p* = 0; Day 21, *p* = 0.001; Day 28, *p* = 0.003; Day 60, *p* = 0.010). Additionally, a significant difference was observed between Day 0 and Day 60 in the reference cream patients (*p* < 0.05, *p* = 0.008).

Regarding melanin, no statistically significant variation was observed between the two interventions (Figure 8D).

**Figure 9:**
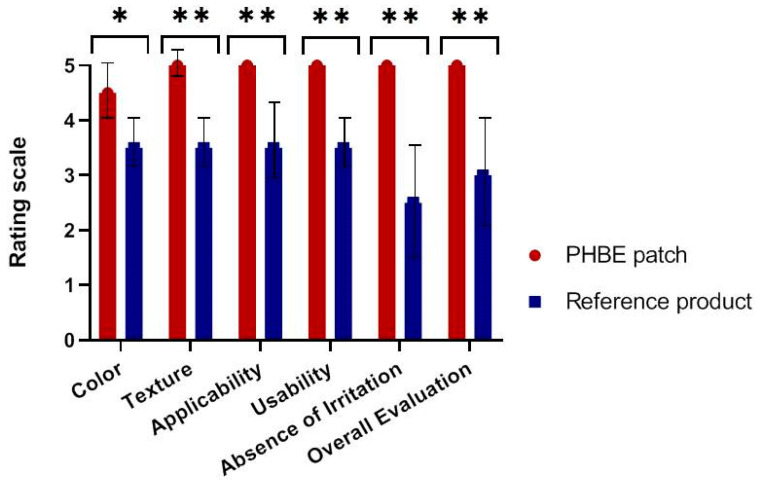
Patients’ evaluation of the two interventions (PHBE patch and reference cream) based on their main characteristics (color, texture, applicability, ease of use, absence of irritation, and overall evaluation). Significant differences were observed between the two interventions (* *p* < 0.05, ** *p* < 0.01), with patients preferring the PHBE patch over the reference cream.

All patients filled out a questionnaire in which they were asked to comment on their experience using the PHBE patch or the reference cream (Figure 9). A content analysis revealed that in terms of color, texture, applicability, ease of use, absence of irritation, and overall evaluation, the patients preferred the PHBE patch over the reference cream. Additionally, the PHBE patch scored highly on all intervention characteristics, except for the color (Figure 9). Significant differences were observed between the two interventions concerning all characteristics (color, *p* = 0.015; texture, *p* = 0.004; applicability, *p* = 0.002; ease of use, *p* = 0.002; absence of irritation, *p* = 0.002; overall evaluation, *p* = 0.002).

The obtained results are in accordance with recent research data on barrier-forming products in radiotherapy and corroborate the assumed principle of prophylactic superficial skin protection for the reduction or even prevention of radiation dermatitis [50,51,52,53]. The main limitation of the present clinical trial was the small sample size, while another limitation was the subjective scale (VAS) used for measuring pain and itching severity, which could not exclude patient bias. However, a uniform treatment site was attempted, without varied fractionation schedules and patient compliance with prophylactic interventions. Considering the promising results of this preliminary study, a clinical trial with a higher number of volunteer patients should be conducted.

## 4. Conclusions

In this study, the efficiency of an optimized PHBE micro/nanofibrous patch on preventing or treating acute radiodermatitis in NMSC patients undergoing radiotherapy was assessed in comparison with a commercially available reference cream. The topical application of the PHBE patch significantly contributed to prophylaxis and successful management of acute radiodermatitis throughout the treatment period. Significant beneficial effects were observed on the RTOG scale, TEWL, erythema, hemoglobin concentration, skin texture, and subjective itching and pain experience, while no statistically significant variation between the two interventions was observed for hydration and melanin. Moreover, 1 month after RT, the PHBE patch, in contrast to the reference cream, eliminated skin inflammation, restored the biophysical skin parameters to normal values, and reduced the discomfort of the patients. The PHBE patch was well accepted by all patients and proved statistically effective, without any adverse reaction reported, indicating that the PHBE patch is a safe prophylactic radiodermatitis treatment. Taking into account that numerous clinical studies on systemic and topical treatments have not yet succeeded in providing satisfactory prophylaxis for acute radiodermatitis, the effective application of bioactive micro/nanofibrous non-woven patches loaded with PHBE could offer great potential towards the development of a new generation of anti-inflammatory topical skincare dressings with tunable properties and controlled administration characteristics.

## Figures and Tables

**Table 1:** Characteristics of the patients at baseline.

Characteristics of Patients	PHBE Patch Group (*N* = 6)	Reference Product Group (*N* = 6)	Total Patients (*N* = 12)
**Demographics**			
Median age (interquartile range)—year	86 (80–93)	75 (51–90)	80 (51–93)
Gender—%			
Male	66.7	83.3	75.0
Female	33.3	16.7	25.0
Body mass index—%			
>25 (overweight)	33.3	50	41.7
<25 (healthy weight)	66.7	50	58.3
Fitzpatrick skin type—%			
I	16.7	33.3	25.0
II	50.0	33.3	41.7
III	33.3	33.3	33.3
Unprotected sun exposure—%	100.0	83.3	91.7
Current Smoker—%	0.0	16.7	8.3
**Characteristics of non-melanoma skin cancer (NMSC)**			
Cancer type—%			
Basal cell carcinoma (BCC)	50.0	50.0	50.0
Squamous cell carcinoma (SCC)	33.3	16.7	25.0
Basosquamous carcinoma (BSC)	16.7	33.3	25.0
Tumor size (cm^2^)—%			
>20	50.0	50.0	50.0
<20	50.0	50.0	50.0
Primary—%	83.3	50.0	66.7
Recurring—%	16.7	50.0	33.3
Surgery—%	100.0	66.7	83.3
**Radiation therapy (RT)**			
Total dose (cGy)—%			
5750	50.0	16.7	33.3
5500	33.3	66.7	50.0
4800	16.7	16.7	16.7
Fraction dose (cGy)—%			
250	66.7	50.0	58.3
200/400 ^1^	16.7	0.0	8.3
250/300 ^1^	16.7	16.7	16.7
250/308 ^1^	0.0	16.7	8.3
300/400 ^1^	0.0	16.7	8.3
Fractions—%			
15	0.0	16.7	8.3
22	33.3	66.7	50.0
23	66.7	16.7	41.7

^1^ Combination of fraction doses.

**Table 2:** *p*-values for patients’ baseline characteristics. Values lower than or equal to 5% (*p* ≤ 0.05) indicate a statistical difference.

Characteristics of Patients	*p*-Value
**Demographics**	
Age	0.394
Gender	0.699
Body mass index	0.132
Fitzpatrick skin type	0.818
Sun exposure	0.699
Smoking	0.699
**Characteristics of non-melanoma skin cancer (NMSC)**	
Cancer type	0.818
Tumor size	0.394
Primary or recurring	0.394
Surgery or not	0.394
**Radiation therapy (RT)**	
Total dose	0.310
Fraction dose	0.394
Fractions	0.132

## Data Availability

The data presented in this study are available on request from the corresponding authors.

## Supplementary Materials

The following are available online at https://www.mdpi.com/article/10.3390/cancers13112596/s1. Figure S1: Application of the PHBE patch on a representative patient enrolled in the study. Figures S2 and S3: Images of all patients treated with the PHBE patch and the reference product, respectively, during Days 7, 14, 21, and 28, and 1 month after RT (Day 60). Figures S4 and S5: Antera 3D images of all patients treated with the PHBE patch and the reference product, respectively, during Days 7, 14, 21, and 28, and 1 month after RT (Day 60).
