# Supplementary material for: Management of Acute Radiodermatitis in Non-Melanoma Skin Cancer Patients Using Electrospun Nanofibrous Patches Loaded with Pinus halepensis Bark Extract
Source: Cancers (Basel). 2021 May 26;13(11):2596. doi: 10.3390/cancers13112596 (PMC8199239; doi:10.3390/cancers13112596)
Supplement: Supplementary file 1 [file cancers-13-02596-s001.zip › cancers-1214905-supplementary.pdf]

Management of Acute Radiodermatitis in Non-Melanoma Skin Cancer Patients Using Electrospun Nanofibrous Patches Loaded with *Pinus halepensis* Bark Extract

Aikaterini Kyritsi [1], Stefanos Kikionis [2], Anna Tagka [3], Nikolaos Koliarakis [3], Antonia Evangelatou [3], Panagiotis Papagiannis [3], Alexandros Stratigos [3], Vangelis Karalis [1], Paraskevas Dallas [1], Andreas Vitsos [1], Efstathia Ioannou [2], Vassilios Roussis [2,*] and Michail Rallis [1,*]

[1] Section of Pharmaceutical Technology, Department of Pharmacy, School of Health Sciences, National and Kapodistrian University of Athens, Panepistimiopolis Zografou, Athens 15784, Greece; katerinakyr18394@gmail.com (A.K.); vkaralis@pharm.uoa.gr (V.K.); dallas@pharm.uoa.gr (P.D.); avitsos@yahoo.gr (A.V.)

[2] Section of Pharmacognosy and Chemistry of Natural Products, Department of Pharmacy, School of Health Sciences, National and Kapodistrian University of Athens, Panepistimiopolis Zografou, Athens 15771, Greece; skikionis@pharm.uoa.gr (S.K.); eioannou@pharm.uoa.gr (E.I.)

[3] First Department of Dermatology-Venereology, National and Kapodistrian University of Athens, Andreas Syggros Hospital, 5 Ionos Dragoumi Str., Athens 11621, Greece; annatagka@gmail.com (A.T.); nkoliarakis@yahoo.gr (N.K.); antevagelatou@gmail.com (A.E.); PPaPagi@med.uoa.gr (P.P.); alstrat2@gmail.com (A.S.)

* Correspondence: roussis@pharm.uoa.gr (V.R.); rallis@pharm.uoa.gr (M.R.); Tel.: +30-210-7274592 (V.R.); +30-210-7274699 (M.R.)

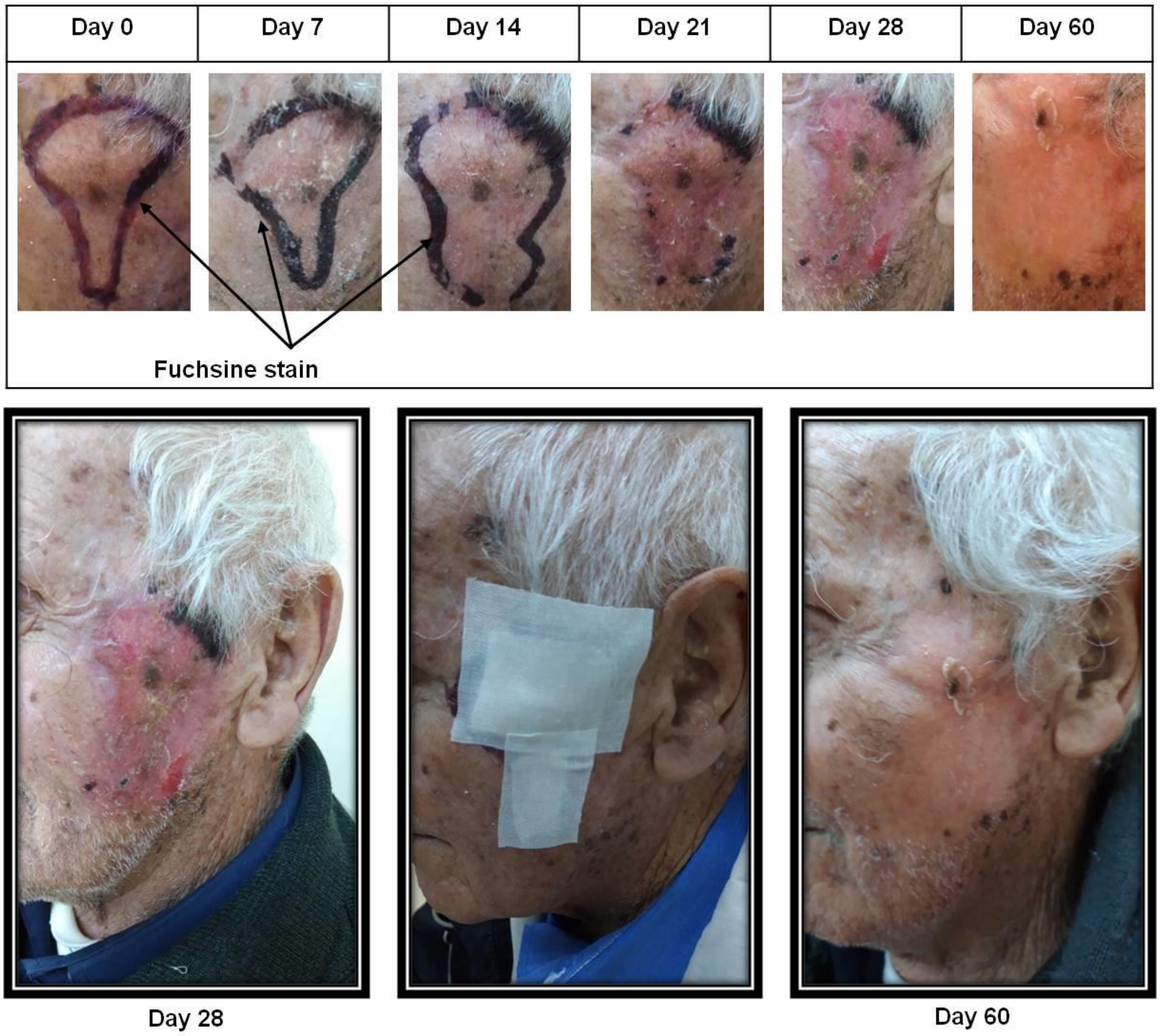

Figure S1. Application of the PHBE patch on a representative patient enrolled in the study.

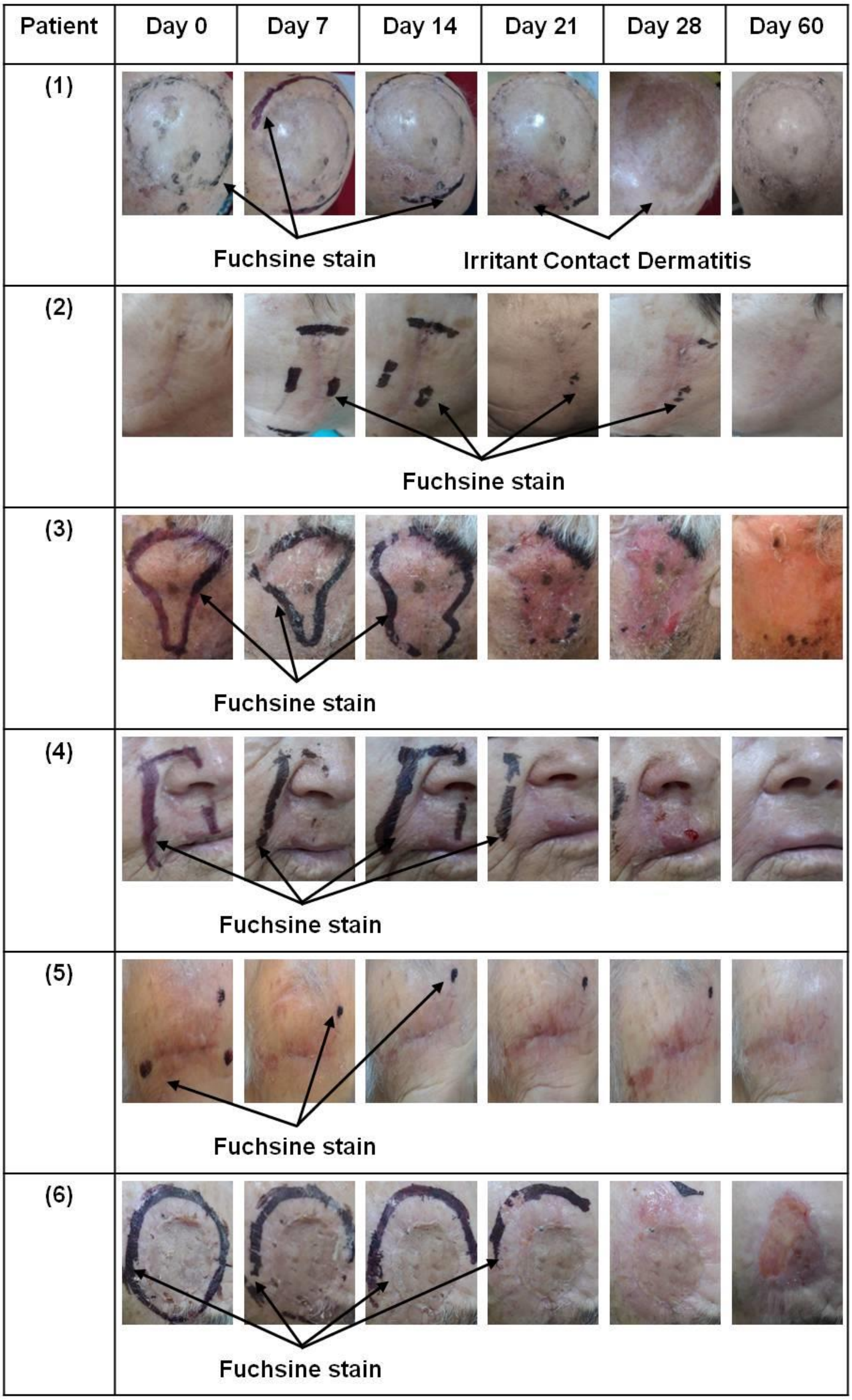

Figure S2. Images of all patients treated with PHBE patch, during days 7, 14, 21, and 28, and one month after RT (day 60).

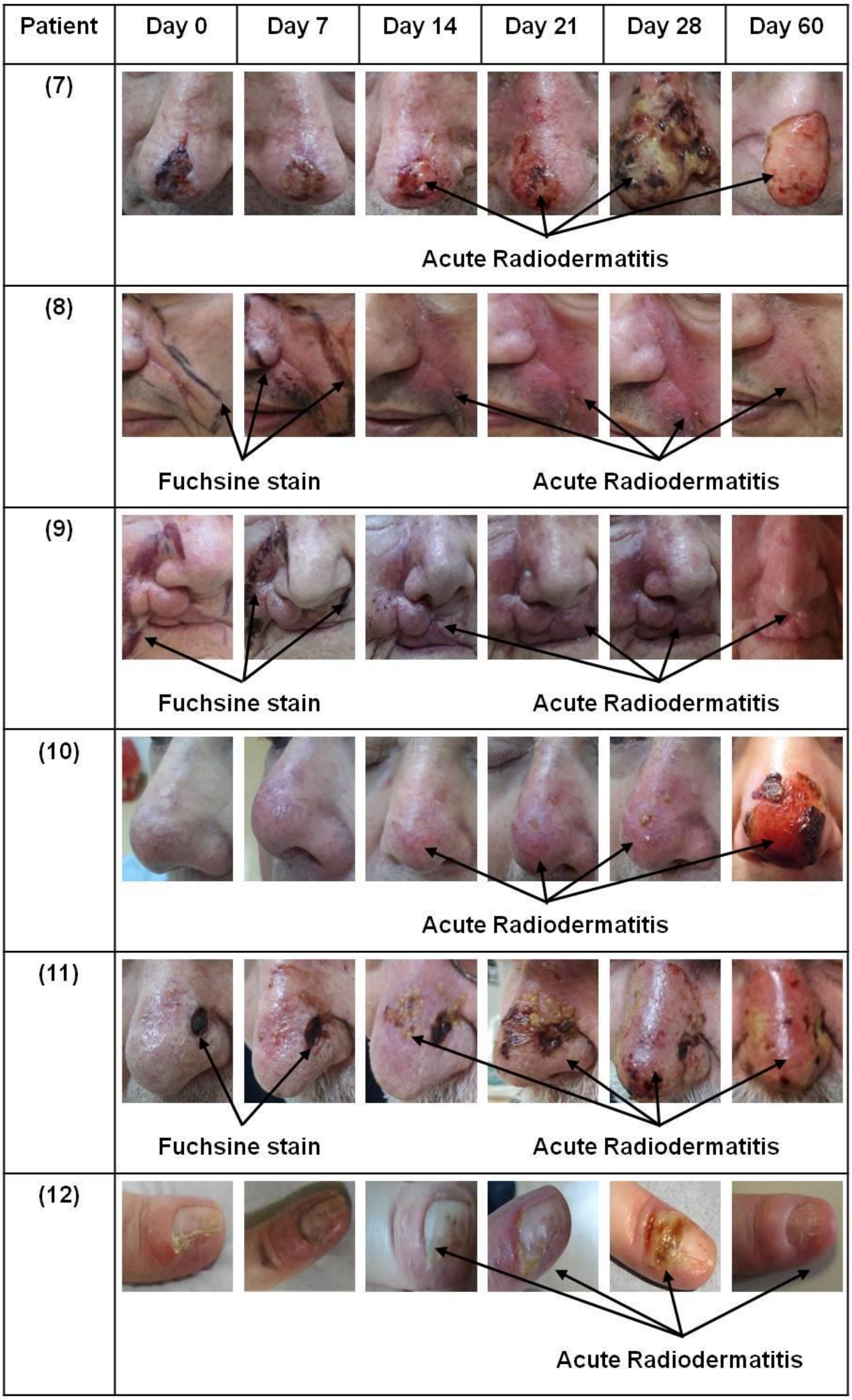

Figure S3. Images of all patients treated with the reference product, during days 7, 14, 21, and 28, and one month after RT (day 60).

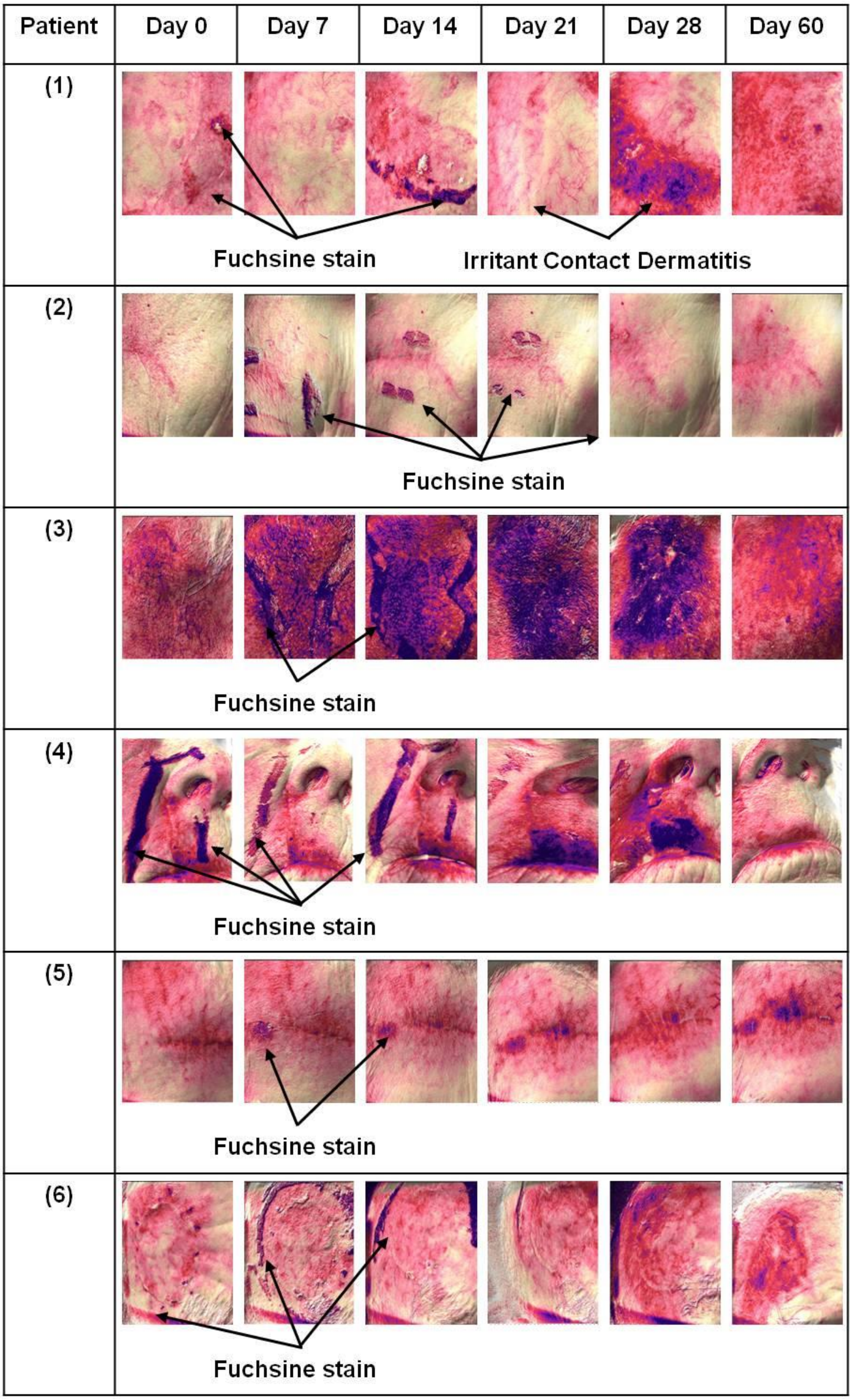

Figure S4. Antera 3D images of all patients treated with PHBE patch, during days 7, 14, 21, and 28, and one month after RT (day 60).

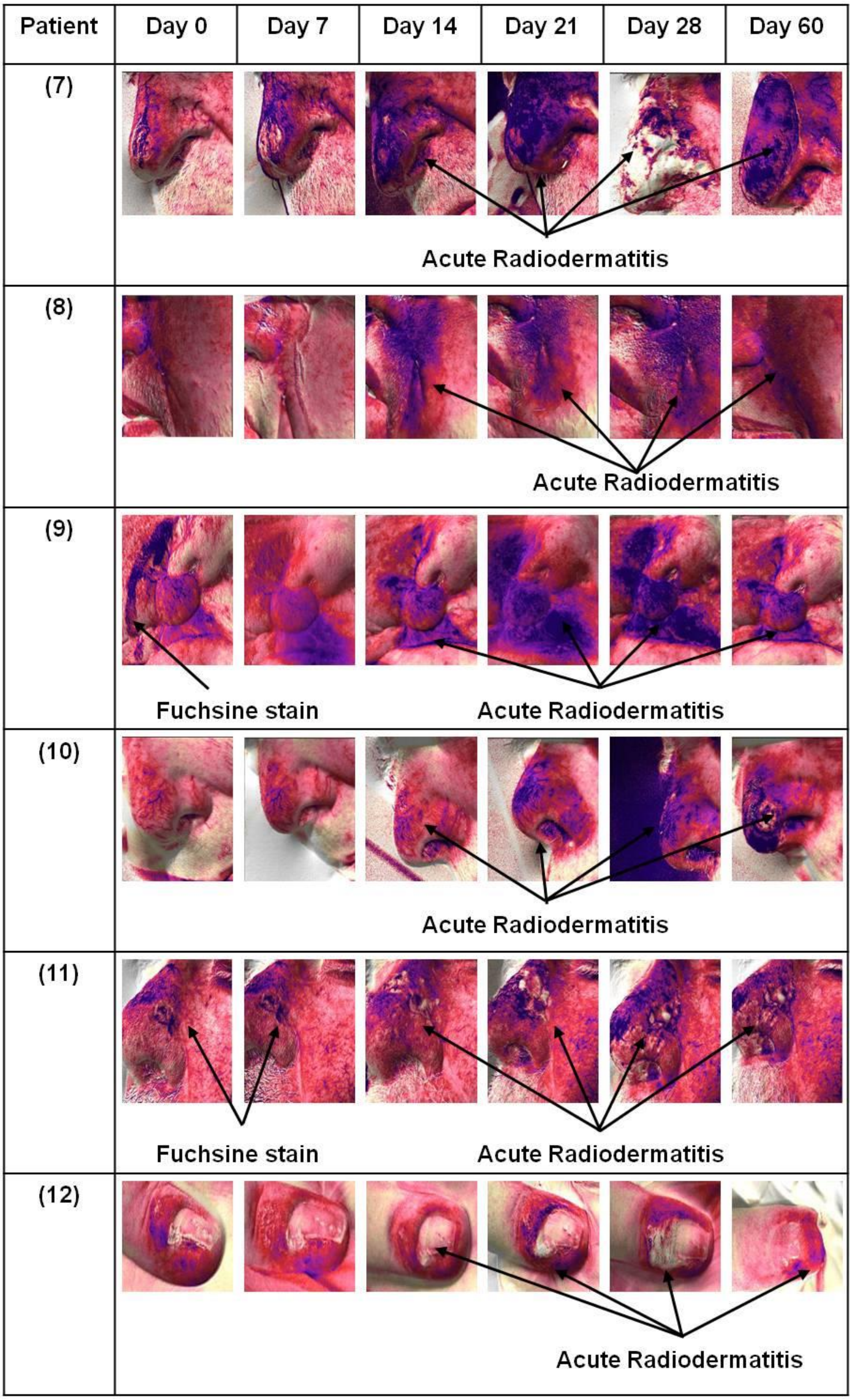

Figure S5. Antera 3D images of all patients treated with the reference product, during days 7, 14, 21, and 28, and one month after RT (day 60).
